# Evidence for Ancestral Programming of Resilience in a Two-Hit Stress Model

**DOI:** 10.3389/fnbeh.2017.00089

**Published:** 2017-05-11

**Authors:** Jamshid Faraji, Nabiollah Soltanpour, Mirela Ambeskovic, Fabiola C. R. Zucchi, Pierre Beaumier, Igor Kovalchuk, Gerlinde A. S. Metz

**Affiliations:** ^1^Canadian Centre for Behavioural Neuroscience (CCBN), University of LethbridgeLethbridge, AB, Canada; ^2^Faculty of Nursing and Midwifery, Golestan University of Medical Sciences (GUMS)Gorgan, Iran; ^3^Department of Anatomical Sciences, Babol University of Medical SciencesBabol, Iran; ^4^Department of Cell Biology, Institute of Biological Sciences, Campus Darcy Ribeiro, University of Brasilia (UnB)Brasilia, Brazil; ^5^CanAlt Health LaboratoriesConcord, ON, Canada; ^6^Department of Biological Sciences, University of LethbridgeLethbridge, AB, Canada

**Keywords:** multigenerational prenatal stress, ancestral stress, stress resiliency, skilled reaching, silent cerebral ischemia, mini stroke, two-hit theory

## Abstract

In a continuously stressful environment, the effects of recurrent prenatal stress (PS) may accumulate across generations and alter stress vulnerability and resilience. Here, we report in female rats that a family history of recurrent ancestral PS facilitates certain aspects of movement performance, and that these benefits are abolished by the experience of a second hit, induced by a silent ischemia during adulthood. Female F4-generation rats with and without a family history of cumulative multigenerational PS (MPS) were tested for skilled motor function before and after the induction of a minor ischemic insult by endothelin-1 infusion into the primary motor cortex. MPS resulted in improved skilled motor abilities and blunted hypothalamic-pituitary-adrenal (HPA) axis function compared to non-stressed rats. Deep sequencing revealed downregulation of miR-708 in MPS rats along with upregulation of its predicted target genes *Mapk10* and *Rasd2*. Through miR-708 stress may regulate mitogen-activated protein kinase (MAPK) pathway activity. Hair trace elemental analysis revealed an increased Na/K ratio, which suggests a chronic shift in adrenal gland function. The ischemic lesion activated the HPA axis in MPS rats only; the lesion, however, abolished the advantage of MPS in skilled reaching. The findings indicate that MPS generates adaptive flexibility in movement, which is challenged by a second stressor, such as a neuropathological condition. Thus, a second “hit” by a stressor may limit behavioral flexibility and neural plasticity associated with ancestral stress.

## Introduction

Prenatal stress (PS) is one of the most critical determinants of health and disease (Cottrell and Seckl, 2009). PS is associated with poor health later in life, including a higher risk of hypertension and cardio- and cerebrovascular disease (Igosheva et al., 2007; van Dijk et al., 2012). Moreover, stress has been recognized as a critical variable in stroke risk and recovery (Kirkland et al., 2008; Dunn et al., 2011; Walker et al., 2014). Indeed, stress represents a critical regulator of metabolic and cardiovascular function and is the cause of hypertension, the number one risk factor for stroke (Kulkarni et al., 1998). Experimental evidence revealed that PS is associated with poor stroke outcome (Wang et al., 2015). Accordingly, PS impedes recovery after ischemic lesion with associated transcriptomic changes (Zucchi et al., 2014).

PS has long-term consequences on fetal endocrine function involving the hypothalamic-pituitary-adrenal (HPA) axis (Harris and Seckl, 2011). Prenatal programming of HPA axis properties may explain the large variability in recovery among stroke patients (Nudo, 2007). In prenatally stressed individuals, the experience of a stroke may represent a second hit to the dysregulated HPA axis and challenge neural plasticity with inflammation, hypoxia-ischemia, neuronal death and neurodegenerative processes (Craft and Devries, 2009). In addition, patients with stroke frequently report that they experience difficulties in coping with stress (Green and King, 2007) or experience emotional and psychological distress (Duric et al., 2016), which may further aggravate the symptoms or impede the recovery process.

The consequences of PS are potentially carried forward through several generations of offspring (Dias and Ressler, 2014; Gapp et al., 2014a). Through epigenetic mechanisms, such as microRNA (miRNA) expression, PS can alter stress response, motor function and health trajectories of subsequent generations, with pronounced changes when challenged with recurrent stress (Ward et al., 2013; Yao et al., 2014; Zucchi et al., 2014). As opposed to stress vulnerability, a continuously stressful environment may favor the development of stress tolerance or resilience. Repeated generational PS exposure gradually promotes the formation of new behavioral traits (Ambeskovic et al., 2017) and coherence between brain areas (Skelin et al., 2015). The extent to which cumulative effects of multigenerational PS (MPS) can promote stress resilience, especially in generally more resilient females, has not yet been studied.

Here, we use a rat model of silent focal ischemia, which due to its covert symptoms provides an ideal framework to study stress resilience (Faraji et al., 2011, 2012). We examined if the recurrent impact by MPS across three generations can influence focal cerebral ischemia outcomes in the F4 offspring. We hypothesized that MPS and ischemia represent two “hits” of stress that will compromise stress resilience (Knudson, 1971; Olson et al., 2015). The results provide new insights into the origins of stress vulnerability and resilience and individual variability in stroke outcome and brain plasticity.

## Materials and Methods

### Animals

Thirty-four adult female Long-Evans rats, weighing 295–335 g at the beginning of the experiment and raised at the local breeding colony at the Canadian Centre for Behavioral Neuroscience, were used in this study. The animals were housed in pairs or groups of three under a 12:12 h light/dark cycle with light starting at 07:30 AM and temperature set at 22°C. All of the testing and training was performed during the light phase of the cycle at the same time of day.

The animals received an unlimited supply of water. Animals were food-restricted 1 week prior to baseline training, and were maintained at approximately 90% of their initial body weight. All animals were weighed and then fed 3 h after completion of daily behavioral test or training sessions. Daily weighing of animals ensured adequate monitoring of food consumption.

All procedures and experimental manipulation including animal handling, food restriction, blood sampling, gestational stress, experimental focal ischemia surgery and behavioral testing were performed under protocols approved by the Animal Care Committee of the University of Lethbridge in compliance with the guidelines of the Canadian Council on Animal Care.

### Experimental Design

Rats derived from multigenerationally stressed and non-stressed lineages (F4 generation) bred under controlled conditions were trained in a skilled forelimb reaching task (at least 10 days) and the ladder rung walking task (1 day) prior to cortical ischemic lesion. Motor performance was videorecorded on the day prior to lesion (Baseline) for movement analysis. The animals were then divided into four groups: Sham (*n* = 8), Stroke-only (*n* = 8), Stress-only (*n* = 8) and Stress + Stroke (*n* = 10). In stroke-only and stress + stroke groups, cortical ischemic lesion was induced by endothelin-1 (ET-1) infusion into the primary motor cortex (M1) of the dominant hemisphere, i.e., on the side contralateral to the paw preferred in reaching. Non-lesion groups of rats received a sham surgery in which all procedures except for the trephination and the injection of ET-1 were received. All animals were allowed to recover for 2–3 days before testing began.

After recovery from surgery, rats completed 16 days of skilled forelimb reaching testing until post-lesion day 19 and 2 days of assessment in skilled walking on post-lesion days 20–21. After behavioral assessments were completed, animals were euthanized for histological and biological analysis.

### Gestational Stress Procedures

To test the cumulative effects of adverse prenatal experience, timed-pregnant rats (F0–F3) in each generation were stressed daily from gestational day 12 to day 18. Two stressors, restraint of the body for 20 min (Metz et al., 2005a) and forced swimming in water at room temperature for 5 min (Metz et al., 2001; Vyas et al., 2002) were applied once daily in a semi-random sequence, either in the morning (8:30 AM) or in the afternoon (4:30 PM) hours.

Two different lineages of rats were bred under standardized conditions. In the multigenerational stress lineage, the pregnant parental (F0) generation, their pregnant F1 daughters, F2 granddaughters and F3 great-granddaughters were stressed during pregnancy (Erickson et al., 2014; Yao et al., 2014; Skelin et al., 2015). A lineage of yoked controls was bred with each generation. A maximum of two female offspring per litter were randomly selected to be included in the experiments. Each experimental group included offspring therefore from at least four different litters.

### Skilled Forelimb Reaching Task

Skilled forelimb reaching assessment was based on earlier descriptions (Metz and Whishaw, 2000; Faraji et al., 2011). Each training and test session required the rats to reach for 20 banana-flavored food pellets (45 mg, Bioserv Inc., Frenchtown, NJ, USA) in a transparent Plexiglas chamber (40 cm × 45 cm and 13.1 cm wide). Baseline training was considered complete once the success rates reached asymptotic levels for three consecutive days. Quantitative analysis of reaching performance included the percentage of successes and reaching attempts according to earlier descriptions (Metz and Whishaw, 2000).

### Skilled Walking and Paw Placement

The ladder rung walking task was used to determine skilled fore- and hind limb use (Metz and Whishaw, 2002). Animals were trained to cross a custom-made 1 m-long horizontal ladder rung walking task with irregularly spaced round metal rungs. The rungs were arranged at random distances ranging from 0.5 cm to 5 cm. The same pattern was maintained for all training and test sessions. Animals were trained in four trials to cross the ladder on the day prior to the actual testing. Each test session consisted of three trials per animal, during which the animals’ performance was videorecorded from a ventrolateral perspective for further movement analysis.

A 7-category rating system was used to determine the type of hind foot placement on the rung for hind limbs according to earlier descriptions (Metz and Whishaw, 2002). A foot fault score of 0 was given for a total miss whereas a score of 6 indicated a correct limb placement with full weight support. The number of errors per step in each crossing was also calculated (Metz and Whishaw, 2002). Scores and the mean number of hind limb errors per step were averaged across three trials.

### Blood Samples

Blood samples were taken a day prior to and a day after cortical lesion. All samples were collected in the morning hours between 9:00 and 11:00 AM (Metz et al., 2005a). Rats were transported individually to the surgical suite and anesthetized with 4% isoflurane for 2–3 min as approved by the Institutional Animal Care Committee. From the tail vein, 0.7 ml of blood was collected using a heparinized butterfly catheter. Plasma was obtained by centrifugation at 7000 rpm for 5 min. The plasma samples were stored at 20°C until analyzed for corticosterone (CORT) concentration using commercial radioimmunoassay kits (Coat-A-Count, Diagnostic Products Corporation, Los Angeles, CA, USA).

### Cortical Focal Ischemic Lesion Induced by Endothelin-1

Procedures for focal silent ischemic lesions using unilateral endothelin-1 (ET-1) infusion in the primary motor cortex (M1) were modified from Faraji et al. (2012). Briefly, rats were anesthetized using 1.5% isoflurane inhalation. A midline incision was made in the scalp and periosteum, and two injections of ET-1 (Sigma–Aldrich, St. Louis, MO, USA) were made (AP: +1.60, +2.20; ML: ±3, ±2.50; DV: −2, −2; 175 pmol; 0.12 μl; 0.3 μl/min) on the side contralateral to the paw preferred for skilled reaching. ET-1 was delivered through a 23-gauge cannula attached to a Harvard infusion pump (Model 22). The cannula was left in place for 5 min after each injection to allow for ET-1 diffusion. The scalp was sutured and the recovery of the animals was monitored. Post-surgical care included the administration of buprenorphine HCl (0.05 mg/kg; Reckitt Benckiser Healthcare Ltd. UK) as an analgesic. Sham-operated animals received all surgical procedures except skull trephination and ET-1 injection.

### Histology and Morphological Analyses

Rats were euthanized with an overdose of sodium pentobarbital (300 mg/kg i.p.) and perfused intracardially with saline (0.9%; 200 ml/rat) followed by 4% paraformaldehyde (PFA; 200 ml/rat). Brains were removed, post-fixed for 24 h in 4% PFA and cryoprotected in 30% sucrose and 4% paraformaldehyde at 4°C for coronal sectioning (40 μm) and staining. Every fourth section was mounted on glass slides and stained with cresyl violet. The stained sections were examined under a microscope (Zeiss, Jena, Germany) and images were captured using an AxioCam camera (Zeiss, Jena, Germany) for histological analysis and presentation. From the coronal sections, measures were made of cortical thickness, neuronal density, brain volume and lesion size in both hemispheres of all groups. The experimenter was blind to the experimental groups.

#### Cortical Thickness

Briefly, three points (central, lateral and ventrolateral) on nine coronal sections (AP 4.20, 3.70, 2.70, 2.20, 1.70, 1.60, 1.20, 0.48, and −0.26 mm) from each brain were selected based on Paxinos and Watson (1997). Therefore, the most rostral section measured was located at ~4.20 mm anterior to Bregma and the most caudal section at ~ −0.26 mm posterior to Bregma. For each point, a vector was considered from the tangent of the outer edge to the inner edge of the cortex. ImageJ software 1.47b^1^ (NIH, Bethesda, MD, USA) was used to record up to eight measurements of cortical thickness from each coronal section, three from each hemisphere.

#### Neuronal Density (Densitometry)

Neuronal density analysis (quantitative cytoarchitectonics) was performed using ImageJ 1.47b based on earlier descriptions (Moon et al., 2009). A calibration was made by a step tablet for optical density prior to the analysis of density. Two approximate planes (between planes 8 and 15; 3.20 mm and 0.70 mm anterior to Bregma; Paxinos and Watson, 1997) of stained sections in each brain were selected. Two central and lateral regions of interest (ROIs) were determined within the somatosensory cortical regions, adjacent to the lesion area of each hemisphere. Both left and right ROIs included the same cortical regions and nearly all cortical layers. An absolute gray value index (GVI) or the average gray value within the ROIs was separately measured for each region.

#### Brain Volume Analysis

For each animal, a set of 37 cross sections of the whole brain except olfactory bulb and cerebellum stained with cresyl violet was considered for volumetric analysis. Images of the stained sections were captured using an AxioCam (Zeiss, Jena, Germany). The most rostral section measured was located at 5.20 mm anterior to Bregma and the most caudal section at −7.30 mm posterior to Bregma. For each tissue section, the contours of the bilateral hemispheres were traced and their areas were measured using ImageJ 1.47b. Brain volume averages were calculated by dividing the sum of measures obtained from each brain by the total number of sections (Shrunk Brain Area in mm^2^). The approximate volume of the brain (Shrunk Brain Volume in mm^3^) was determined by multiplying the total area in mm^2^ by both the thickness of each slice (40 μm) and the sampling interval (3):

$$Shrunk\;Brain\;Volume\;\left(mm^{\text{3}}\right)\text{ = }Area\;mm^{\text{2}}\text{× 0.04 × 3}$$

Estimated measures of brain volume in the present experiment did not contain olfactory bulb and cerebellum.

#### Lesion Size Analysis

Cortical lesion extent in each ischemic rat was estimated according to the Cavalieri method (Schmitz and Hof, 2005). Five images were captured, corresponding approximately to 0.48, 1.00, 1.60, 2.20 and 3.20 mm relative to Bregma. A systematic sampling grid with an area per point of 20,000 pixels was randomly thrown over each image captured under 1× magnification and the numbers of points hitting the intact motor cortex area were counted. Grids were generated using ImageJ 1.47b (NIH, Bethesda, MD, USA). The total number of hits in each rat was then divided by the average number of hits obtained by three control rats. The complement proportion was used as the percentage motor cortex lesion estimate (Faraji et al., 2012). This assessment was intended to indicate an overall difference in cortical damage in different experimental groups.

### Hair Trace Elemental Analysis

Hair elemental analysis was based on inductively coupled plasma mass spectrometry (ICPMS) to examine the level of sodium/potassium (Na/K) ratio as an indicator of chronic alterations in adrenal gland activity due to ancestral stress (Ambeskovic et al., 2013). Approximately 0.6–0.8 g of abdominal and back hair was collected with scissors post-mortem. To control for metal trace contamination, fabric was cut with the same pair of scissors and analyzed as separate samples.

Hair sample analysis was performed by CanAlt Health Laboratories (Concord, ON, Canada). Hair samples were cut into small pieces using clean stainless steel scissors. About 300 ± 5 mg were transferred into tarred, labeled centrifuge tubes, and the exact weight was recorded. To each sample digestion tube, 3.0 ml of reagent-grade nitric acid (HNO_3_) was added. Samples were incubated for 25 min. Samples were then subjected to acid microwave digest in order to stabilize the elements of interest. The digestate solution was analyzed for amounts of the mineral element and trace metals by ICPMS. Sample results were quantified by comparison with calibration solutions of known concentrations (Ambeskovic et al., 2013).

#### miRNA and mRNA Deep Sequencing

A subgroup of animals (*n* = 3 per group) received an overdose of pentobarbital (Euthansol 100 mg/kg; CDMV Inc., Quebec, QC, Canada). After vital signs discontinued animals were rapidly decapitated. Brains were rapidly removed, dissected and flash-frozen for miRNA and mRNA analysis. Using TRI Reagent Solution total RNA was extracted from the frontal cortex. miRNA expression analysis used a Illumina GAIIx genomic analyzer (Illumina Inc., San Diego, CA, USA). Briefly, base calling and demultiplexing was completed using CA SAVA 1.8.1 software pipeline with default settings. FastQC software was used to examine short read quality. Adapters were trimmed using cutadapt software^2^. After trimming FastQC quality check was performed. Standalone MicroRazerS version 1.0 (Emde et al., 2010) was used to preform miRNA detection and counting. The sequence and annotation information reference for rat-RNOR 5.0 (Ensemble) from iGENOME were used (Illumina)^3^.

### Statistical Analysis

Statistical analyses were performed using SPSS 16.0 (SPSS Inc., Armonk, NY, USA). Behavioral data were analyzed using two-way ANOVA (with pre-stress and ischemia as the main factors), followed by *post hoc* (Tukey HSD) analysis to adjust for multiple comparisons between different groups. Dependent variables in the skilled reaching task (success percent and number of attempts), and in the ladder rung walking task (foot placement fault and number of errors) were averaged and analyzed for both pre-ischemic and post-ischemic sessions. Also, values of *η*^2^ = 0.14, 0.06 and 0.01 were considered for large, medium and small effects, respectively. Elemental data were analyzed in parts per million (ppm); however, the graph is shown in percent (%) content change. For miRNA and mRNA data, statistical analysis used the DESeq Bioconductor package (Anders and Huber, 2010). To test for correlations between functional and structural measures Spearman’s rank correlation coefficients were determined. Differences in between-group and within-group comparisons were also assessed with independent and dependent samples *t*-tests for both behavioral and histological data, with *p* < 0.05 set as the significance level. All data are presented as mean ± standard error of the mean.

## Results

### Cumulative Ancestral Stress Programs the Stress Response

#### Plasma CORT Levels

**Figure 1:**
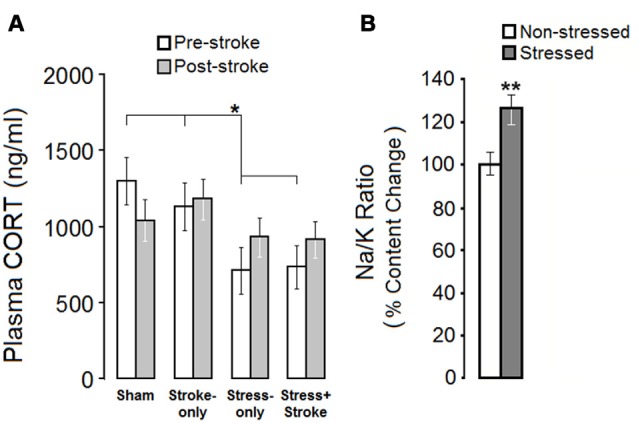
**Mutligenerational stress and cortical ischemic lesion alter hypothalamic-pituitary-adrenal (HPA) axis activity. (A)** Multigenerationally stressed rats showed reduced levels of corticosterone (CORT) prior to focal cortical ischemia when compared with non-stressed rats. No between-group difference was observed in the post-ischemic measures of circulating CORT. **(B)** Multigenerational prenatal stress (MPS) elevated the Na/K ratio in hair, which reflects chronic adrenal gland malfunction. Asterisks indicate significant differences: *p < 0.05, **p < 0.01.

Plasma CORT levels revealed a significant Group effect before (SHAM 1301.48 ± 157 ng/ml; STROKE-only 1132.37 ± 157 ng/ml; STRESS-only 711.27 ± 157 ng/ml; STRESS + STROKE 735.67 ± 141 ng/ml; *F*_(3,30)_ = 5.07 *p* = 0.046; *η*^2^ = 0.38), but not after ischemic events (*p* = 0.06; Figure 1A). A dependent samples *t*-test comparison of pre- and post-ischemic levels of CORT in each group revealed no significant differences between these time points (all *p* > 0.05). Notably, STRESS-only and STRESS + STROKE groups showed lower CORT levels than SHAM or STROKE-only groups before the ischemic event (Figure 1A). Ischemia did not significantly change the levels of circulating CORT in STROKE-only and STRESS + STROKE groups, but the effect of stress compared to non-stress groups disappeared.

#### Hair Trace Element Content Levels

Sodium/potassium (Na/K) ratio was used as an indicator of chronic alterations in adrenal gland activity. An independent sample *t*-test revealed a significant increase in the Na/K ratio in stressed animals before ischemia (Stressed, 0.107 ± 0.04 ppm vs. Non-stressed, 0.082 ± 0.007 ppm; *t*_(13)_ = 4.308, *p* = 0.007; Figure 1B). The stress group showed higher content levels of sodium (631 ± 69) in comparison to controls (581 ± 43). Thus, ancestral stress compromised adrenal gland function and altered circulating CORT levels.

### Cumulative Ancestral Stress Supports Skilled Reaching Ability

#### Reaching Success (Pre-Ischemia)

**Figure 2:**
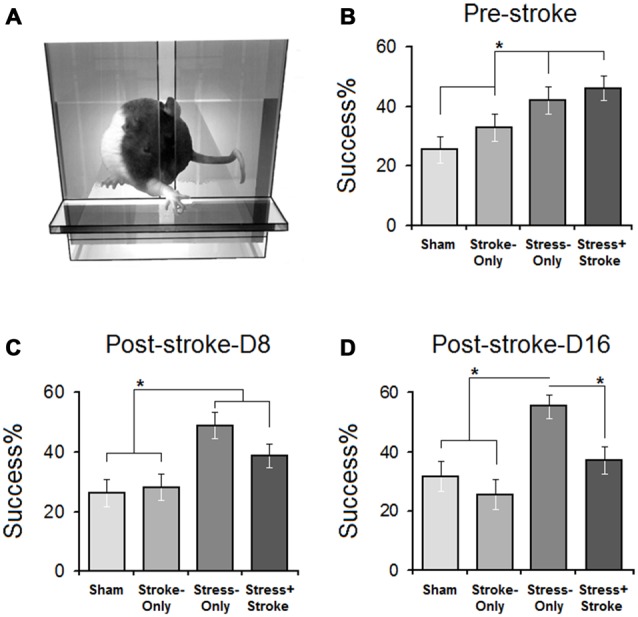
**Improved skilled reaching performance in stressed rats is offset by cortical ischemic lesion. (A)** Illustration of a skilled reaching apparatus. Rats were required to reach and retrieve food pellets with the forelimb contralateral to the ischemic lesion. **(B)** Pre-ischemic reaching success showed that multigenerational PS promoted reaching success compared to non-stressed (stroke-only and stress + stroke) animals. Post-ischemic acute **(C)** and chronic measures **(D)** of reaching success showed significant differences between groups on days 1–8 and 9–16. Reaching success on day 8 in STRESS-only and STRESS + STROKE groups was significantly higher than other groups. Note that ischemic lesion in the stressed animals abolished the advantage of MPS in reaching success. Asterisks indicate significant differences: *p < 0.05, ANOVA.

Assessment of skilled reaching was performed in a transparent Plexiglas chamber (Figure 2A). There was a significant effect of Group (SHAM: 25.5 ± 4.51%, STROKE-only: 32.93 ± 4.47%, STRESS-only: 42.12 ± 4.63%, STRESS + STROKE: 46.10 ± 4.03%; *F*_(3,30)_ = 8.36, *p* = 0.05; *η*^2^ = 0.47). Success rates in STRESS-only and STRESS + STROKE rats were significantly higher than in SHAM and STROKE-only rats (all *p* < 0.05; *Post hoc* Tukey HSD; Figure 2B).

#### Number of Reaching Attempts (Pre-Ischemia)

There was a significant Group effect (SHAM: 88 ± 6.28, STROKE-only: 74 ± 6.89, STRESS-only: 43 ± 5.60, STROKE + STRESS: 46 ± 6.71, *F*_(3,30)_ = 11.29, *p* = 0.032; *η*^2^ = 0.66). STRESS-only and STRESS + STROKE groups made significantly fewer attempts (all *p* < 0.05; *Post hoc* Tukey HSD). Thus, multigenerational stress improved skilled reaching performance.

### Improved Skilled Reaching Performance in Stressed Rats Is Abolished by Cortical Lesion

#### Reaching Success (Post-Ischemia)

Reaching success was assessed on days 1–8 (Figure 2C) and 9–16 (Figure 2D) after the ischemia. Acute ischemia resulted in a significant main effect of Group (SHAM: 26.17 ± 4.06%, STROKE-only: 28.2 ± 4.01%, STRESS-only: 48.90 ± 4.06%, STRESS + STROKE: 38.81 ± 4.02%; *F*_(3,30)_ = 6.08, *p* = 0.039; *η*^2^ = 0.47) and Day (*F*_(7,30)_ = 5.91, *p* = 0.044; *η*^2^ = 0.28). At the acute time point, STRESS-only and STRESS + STROKE rats showed significantly higher success rates when compared to SHAM and STROKE-only groups (all *p* < 0.05; *Post hoc* Tukey HSD). Chronic changes also induced a main effect of Group (SHAM: 31.79 ± 4.33%, STROKE-only: 25.62 ± 4.37%, STRESS-only: 55.46 ± 5.10%, STRESS + STROKE: 37.25 ± 4.56%; *F*_(3,30)_ = 12.37, *p* = 0.042; *η*^2^ = 0.39) and Day (*F*_(7,30)_ = 8.22, *p* = 0.040; *η*^2^ = 0.21). STRESS-only rats significantly improved in reaching success compared to STRESS + STROKE rats during the chronic phase (55.46 ± 5.10% vs. 37.25 ± 4.56%; *p* < 0.037; *Post hoc* Tukey HSD). Furthermore, within-subject analysis to compare pre- and post-ischemic changes (effect of Time) in reaching performance also indicated that reaching success in STRESS + STROKE animals was reduced (*F*_(2,26)_ = 11.91, *p* = 0.036) compared with pre-ischemic levels) *p* < 0.041; *Post hoc* Tukey HSD).

#### Number of Reaching Attempts (Post-Ischemia)

There was a significant main effect of Group (SHAM: 76 ± 5.17, STROKE-only: 68 ± 4.09, STRESS-only: 30 ± 4.16, STROKE + STRESS: 34 ± 4.11, *F*_(3,30)_ = 7.20, *p* = 0.041; *η*^2^ = 0.28). STRESS-only rats show non-significantly fewer attempts when compared to STRESS + STROKE animals on acute test sessions (*p* > 0.05; *Post hoc* Tukey HSD. During the post-ischemic chronic phase, however, STRESS-only animals performed significantly fewer attempts compared to STRESS + STROKE animals (19 ± 3.29 vs. 46 ± 4.05; *p* = 0.048; *p* < 0.05; *Post hoc* Tukey HSD). The STRESS-only group performed significantly fewer reaching attempts during the post-ischemic chronic period (30 ± 4.16 vs. 19 ± 3.29; *t*_(7)_ = 4.07, *p* = 0.036). STRESS + STROKE groups showed significantly more attempts during post-ischemic chronic compared to acute trials (46 ± 4.05 vs. 34 ± 4.11; *t*_(9)_ = 6.12, *p* < 0.04). Also, within-subject analysis conducted for reaching attempts in all three time points for each group indicated a significant effect of Time (*F*_(2,26)_ = 6.13, *p* = 0.042) in STRESS + STROKE rats while reaching attempts at the chronic time point were significantly increased (*p* < 0.044; *Post hoc* Tukey HSD). Thus, only animals with both stress and ischemic lesion were less successful than animals that received only stress or ischemia.

### Cumulative Ancestral Stress Reduces Skilled Walking Recovery

All groups improved in skilled walking during post-ischemic assessments. However, STRESS + STROKE rats showed lower accuracy in foot placement when compared to other groups (all *p* < 0.05; *Post hoc* Tukey HSD), thus performing more foot placement errors during the chronic test session.

### Stress and Ischemia Influence Cortical Morphology

#### Cortical Thickness

**Figure 3:**
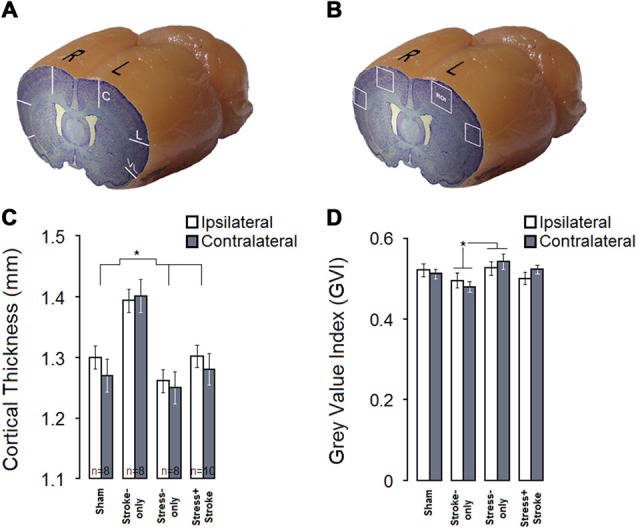
**MPS and cortical ischemic lesion have differential effects on cortical thickness and neural density. (A)** Coronal-sagittal view of a brain illustrating three cortical points (central, lateral, ventrolateral) used for cortical thickness measurements. **(B)** Stroke-only rats show greater cortical thickness compared to other experimental groups in both hemispheres. **(C)** Coronal-sagittal view of a rat brain illustrating bilateral regions of interest for quantitative cytoarchitectonics of absolute gray value index (GVI). White squares indicate four regions of interest (ROIs). **(D)** GVI measures indicated that MPS increased GVI in the contralateral hemisphere, whereas ischemic infarct resulted in reduced GVI in both hemispheres. Asterisks indicate significant differences: *p < 0.05.

Cortical thickness was measured in the central, lateral and ventrolateral portions of both hemispheres (Figure 3A). A significant main effect of Group (*F*_(3,29)_ = 9.66, *p* = 0.000; *η*^2^ = 0.50), but not Hemisphere (*p* = 0.35) or Group by Hemisphere (*p* = 0.72) was observed. Moreover, the effects of Portion (central, lateral, and ventrolateral; *F*_(2,58)_ = 2.50, *p* = 0.000; *η*^2^ = 0.912) and Group by Portion (*F*_(6,58)_ = 2.33, *p* = 0.043; *η*^2^ = 0.195) were significant (Figure 3B). *Post hoc* Tukey HSD analysis also revealed that cortical thickness was largest in STROKE-only animals compared to any other group (all *p* ≤ 0.05).

#### Neuronal Density

Figures 3C,D summarize GVI for neuronal density. Cortical GVI in ipsi and contralateral hemisphere ROIs (Planes 9 and 12; M2–M1 and S1J–S1JO, layers II–V) were measured (Figure 3C). These main findings were confirmed by statistical analysis (Figure 3D). Analysis revealed significant main effects of Group (*F*_(3,30)_ = 4.88, *p* = 0.007, *η*^2^ = 0.328), Hemisphere (*F*_(1,30)_ = 27.63, *p* = 0.000, *η*^2^ = 0.479) and Area (central and lateral; *F*_(1,30)_ = 542.59, *p* = 0.000, *η*^2^ = 0.948). There was also a significant interaction of Group by Hemisphere (*F*_(3,30)_ = 7.72, *p* = 0.001, *η*^2^ = 0.436), but not Group by Area (*p* = 0.902). There was a significant difference between STROKE-only and STRESS-only groups in the GVI measurements (*p* = 0.004, *post hoc* Tukey HSD) because neuronal density in STROKE-only animals was lower than in STRESS-only animals. Correlation analysis revealed no significant relationship between ipsi- and contralateral neuronal density and skilled reaching quantitative scores in either of the groups.

#### Brain Volume Analysis

**Figure 4:**
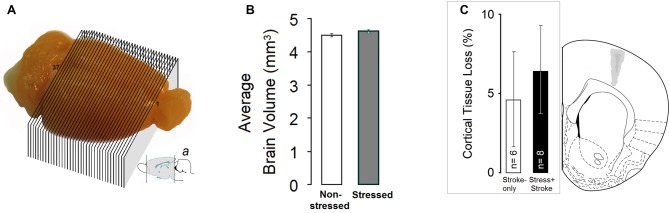
**MPS affects brain volumes.** (**A** and *a*) A set of 37-cross sections of the whole brain except olfactory bulb and cerebellum was considered for volumetric analysis. Sub-panel *a* adopted from Paxinos and Watson (1997). **(B)** Stress-only rats (*N* = 7) showed a non-significant trend towards larger brain volume. **(C)** Tissue loss following focal cortical ischemia in the primary motor cortex (M1). (Left panel) Estimate of the percent damage for stroke-only and stress + stroke rats in M1. (Right panel) Schematic illustration of ischemic lesion extent. Dark and light gray areas indicate the largest and smallest size of ischemic lesion, respectively. Atlas plate (# 11, Bregma 1.70 mm) adopted from Paxinos and Watson (1997).

A set of 37-cross sections of the whole brain except olfactory bulb and cerebellum was considered for volumetric analysis (Figure 4A). Stress overall led to non-significantly larger brain volumes compared to non-stressed groups (*p* = 0.054; Figure 4B). The STRESS-only group had larger volumes in frontal cortex areas compared to any other group (all *p* ≤ 0.05).

#### Lesion Size

The tissue loss induced by ET-1 in the STROKE-only and STRESS + STROKE groups was restricted to the M1 region (Figure 4C). No detectable major tissue damage was observed in secondary motor cortex (M2) or dorsolateral striatum (DLS). Moreover, no noticeable major tissue damage was observed in the central arch of the corpus callosum (CC), except in one STROKE-only animal that showed minor damage to this area, but still remained in the final analysis. One rat from the STRESS + STROKE group with cannula tip dislocation was excluded from the analysis.

The size of the cortical lesions measured from coronal sections ranged from 13.63 ± 2.49% (+1.60 from Bregma) to 8.33 ± 2.56% (+2.20 from bregma) of the intact cortical area. An analysis performed on the percent tissue loss in the motor cortex indicated no significant differences between the ischemic groups (STROKE-only: 4.61 ± 3.20% vs. STRESS + STROKE: 6.38 ± 3.11%; *P* = 0.49, independent samples *t*-test) suggesting that the ET-1 procedure was able to induce a comparable extent of ischemic damage and tissue loss in both groups. There was no correlation between percent lesion size and CORT levels.

#### miRNA and mRNA Profiles

**Figure 5:**
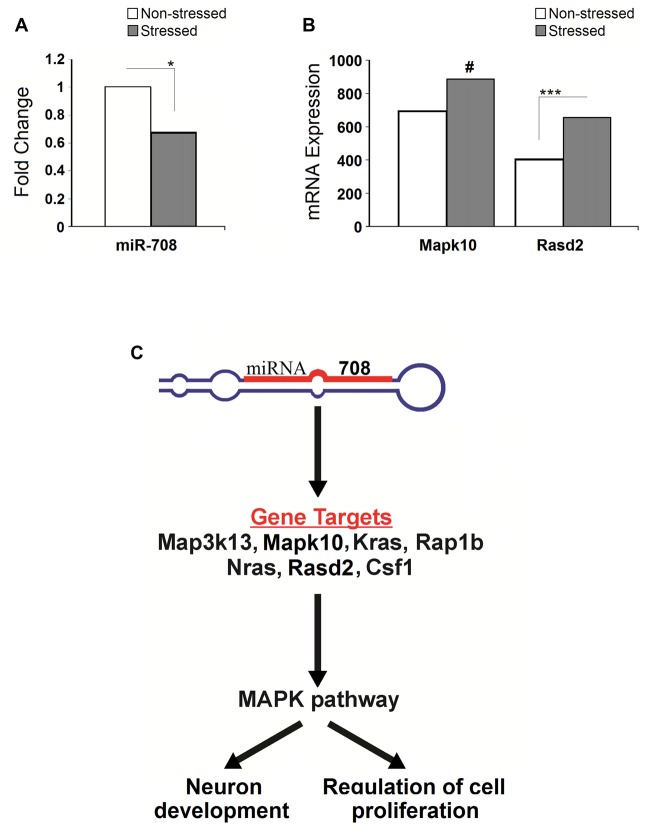
**Stress-induced microRNA and mRNA profiles. (A)** Stress significantly down-regulated expression of the miR-708 in the prefrontal cortex. **(B)** Stress upregulated expression of Rasd2 and Mapk10 genes. **(C)** Illustrative image showing pathways altered by the miR-708. Specifically, miRNA targets five genes (Map3k13, Mapk10, Kras, Rap1b, Nras, Rasd2 and Csf1) which directly affect mitogen activated protein kinase (MAPK) signal pathways, ultimately altering the neuronal development and the regulation of cell proliferation. Asterisks indicate significant differences: *p < 0.05, *** adjusted *p* < 0.001, ^#^indicates near significance.

Compared to non-stressed rats, rno-miR-708-3p was significantly downregulated in the PFC of stressed animals (*p* < 0.05, *n* = 3; Figure 5A). In turn, stressed rats had significantly higher expression of its predicted target gene *Rasd2* compared to non-stressed rats (adj *p* < 0.05). Similarly, stressed rats had upregulated expression of another predicted target gene, *Mapk10* (adj *p* = 0.16, nearly significant), compared to non-stressed animals (Figure 5B). Rno-miR-708-3p potentially targets the *Map3k13, Mapk10, Kras, Rap1b, Nras, Rasd2 and Csf1* mRNAs to potentially regulate neuron development and cell proliferation (Figure 5C).

## Discussion

Stress represents one of the most significant determinants of brain development and neuroplasticity. Here we report that a family history of cumulative ancestral stress has partially beneficial consequences in motor ability, but if challenged by a second hit in later life these benefits may become lost. In female rats, we showed that cumulative effects of MPS promote behavioral flexibility in terms of motor skills and blunts basal HPA axis activity. An increased Na/K ratio of metabolic hair deposits suggests a fundamental, chronic shift in adrenal gland function. By contrast, MPS augmented the HPA response to a second stressor, which was induced by a minor ischemic motor cortex lesion. The hit by a second stressor, a clinically silent ischemic lesion, offset the adaptive benefit in skilled reaching while still promoting functional improvement in skilled walking. The findings further suggest that epigenetic regulation through miRNA may be a central component in stress resilience and vulnerability.

The present data indicate that multigenerational exposure to PS disrupts HPA axis responsiveness, and results in blunted levels of basal circulating CORT levels. Improved motor skill in the presence of lower basal HPA activity in the present study confirms earlier findings that CORT levels and motor ability are negatively correlated (Metz et al., 2005b). Although elevated levels of plasma CORT represent a widely accepted neurohormonal marker of stress (Metz et al., 2005b), both human (Hebert and Lupien, 2007) and animal (O’Connor et al., 2004) data indicate that chronic aversive experiences may lead to reduced responsiveness of the HPA axis to stress and greater risk of psychiatric diseases (Bernatova et al., 2004; Champagne and Meaney, 2006). Hypocortisolism resulting from stress has been linked to hypersensitive HPA axis feedback and blunted plasma CORT levels (Louvart et al., 2005), and it mimics HPA alterations generally reported in post-traumatic stress disorder (PTSD) patients (Yehuda and McEwen, 2004; Cohen et al., 2006). In addition, because the estrous cycle in female rats is closely associated with CORT response (Atkinson and Waddell, 1997), conclusions about HPA-axis activity in the absence of a valid measure of hormonal changes due to the estrous cycle should be drawn with caution.

The consequences of multigenerational stress and focal ischemia are also reflected in morphological changes. The stroke-only rats displayed a significant increase in cortical thickness, likely due to cellular neuroinflammatory responses to the ischemic event (Dirnagl et al., 1999). Interestingly, both ipsilateral and contralateral hemispheres indicated a similar rate of changes in cortical thickness which also has previously been reported in rats (Karl et al., 2010) and humans (Brodtmann et al., 2012). In addition, potential mechanisms may include sex-specific neuroprotective consequences of response to elevated estrogen levels (Hurn and Macrae, 2000; Haast et al., 2012), particularly 17-β estradiol (Liu et al., 2010), through both genomic and non-genomic protective functions (Krause et al., 2006).

Generally, behavioral and morphological consequences of PS seem to be more prominent in males than in females (Mychasiuk et al., 2011). In female rats, a previous study reported both blunted basal CORT and unaffected emotionality after PS (Van den Hove et al., 2013). A reduced impact of PS on behavioral outcomes in females has been linked to sex-specific epigenetic regulation (Van den Hove et al., 2013) and differential endocrine and neurotransmitter responses. The present observation of an increased Na/K ratio in hair, however, suggests that while basal CORT levels were blunted, chronic adrenal gland activity was upregulated (Lawrence, 2010; Schlanger et al., 2010). An increased Na/K ratio may suggest changes in adrenal activity due to an impaired HPA axis feedback loop (Ambeskovic et al., 2013). Thus, the altered Na/K ratio in multigenerationally stressed animals may be an indicator of chronic stress accumulated over generations that alters circulating aldosterone and CORT levels (Hlavacova and Jezova, 2008; Kubzansky and Adler, 2010). Nevertheless, the present findings of superior motor performance indicate behavioral resilience in multigenerationally stressed rats.

HPA axis activity arguably plays a vital role in the formation of stress resilience. For example, offspring of high-licking/grooming mothers display higher hippocampal GR expression and lower DNA methylation of the GR promoter, thus improving HPA axis regulation (Weaver et al., 2004). Thus, stress resilience may be passed on to the second generation (Braga et al., 2012). Accordingly, early life stress in fathers can promote behavioral flexibility in offspring (Gapp et al., 2014b). Moreover, MPS can generate new behavioral traits and a hemispheric dominance shift in the adult F4 generation (Ambeskovic et al., 2017). These changes in multigenerationally stressed offspring parallels simplification of neuronal network processing patterns and improved coherence of brain signaling among cortico-striatal-limbic circuits (Skelin et al., 2015). While the design of the present study does not allow to draw mechanistic conclusions on potential stress resilience, it is possible that epigenetic inheritance of behavioral phenotype (Crews et al., 2012; Zucchi et al., 2012; Gapp et al., 2014a; Yao et al., 2014) may provide a means by which adaptive physiological and emotional responses may be promoted.

Recent findings have provided insights of stress resilience mechanisms involving epigenetic regulation (Wu et al., 2013; Gapp et al., 2014a; Yao et al., 2014). Epigenetic marks generated by PS may potentially be passed on to future generations. Recent studies have pointed out a link between ancestral stress and altered miRNA regulation in the maternal lineage (Zucchi et al., 2013; Yao et al., 2014). Accordingly, our data revealed that MPS downregulates miR-708, which potentially regulates the expression of genes involved in neurotrophin signaling, MAP kinase signaling and axon guidance pathway (Lewis et al., 2005; Xu et al., 2012; Vitucci et al., 2016). miR-708 was shown to be upregulated in hippocampal neurons in response to oxidative stress (Xu et al., 2012). MAPK signaling pathway genes, namely *Map3k13, Mapk10, Kras, Rap1b, Nras, Rasd2* and *Csf1* were predicted bioinformatically to be among main targets of miR-708. Although experimental confirmation at large is still lacking, our study confirmed that miR-708 arguably is a regulator of *Mapk10* and *Rasd2* expression. Moreover, upregulation of miR-708 is associated with increased oxidative stress and subsequent neurodegenerative processes (Bishop et al., 2010). Since miRNAs typically alter the gene expression in mammals through translational inhibition, it is likely that miR-708 negatively regulates the MAPK pathway genes. Our present data indicate that indeed miR-708 may downregulate *Mapk10* and *Rasd2* expression. It can be further inferred that downregulation of miR-708 and upregulation of *Rasd2* and *Mapk10* genes by PS found in our work could contribute to the promotion of neuronal proliferation, likely contributing to the neuronal protection and increased resilience in association With MPS.

Notably, recent work studying biomarkers of resilience or vulnerability to stress in rats demonstrated that the expression of miR-708-5p along with miR-126a-3p was higher at the medial prefrontal cortex (mPFC) of vulnerable rats (Chen et al., 2015). The authors also demonstrated that resilient rats differed from vulnerable rats in the set of multiple blood-circulating miRNAs, namely, reduction in miR-139-5p, miR-28-3p, miR-326-3p, and miR-99b-5p in resilient animals and reduction in miR-24-2-5p, miR-27a-3p, miR-30e-5p, miR-3590-3p, miR-362-3p and miR-532-5p levels in vulnerable animals. This result is consistent with our study demonstrating lower expression of miR-708 in the MPS animals that were also more resilient to stress.

One concept that may potentially explain the present findings is the mismatch hypothesis (Nederhof, 2012; Nederhof and Schmidt, 2012). According to this notion, aversive experiences early in life trigger adaptive processes, such as epigenetic changes, in order to facilitate survival in a stressful environment in later life. The mismatch then occurs if the adaptation to adverse early life experiences is not met by recurrent stress in later life, but by a favorable environment which renders the adaptations superfluous and causes greater risk of disease (Nederhof, 2012; Nederhof and Schmidt, 2012). One assumption for the MPS paradigm may be that the second stressor during pregnancy, at least in the female lineage, reduces the mismatch from one generation to the next. Thus, recurrent PS across generations may promote adaptation of functionally relevant behaviors, such as motor responses that support a flight-or-fight response in a stressful environment.

Although MPS seems to have some benefit in behavioral flexibility in movement strategies, as in the present study, this benefit does not extend to the HPA axis. The vulnerability to physiological disturbances in the stressed lineage is in accordance with other studies that showed that MPS does not protect from stress-associated health complications, such as preterm birth and adverse birth outcomes (Yao et al., 2014), mental health complications (Faraji et al., Submitted) and locomotor hyperactivity (Erickson et al., 2014).

## Conclusion

In spite of some adaptive consequences for motor capacity, the present study supports previous findings that a history of ancestral stress nevertheless has adverse consequences on health outcomes (Yao et al., 2014). To our knowledge, the present study is the first to investigate the influence of ancestral stress on recovery from a brain injury. The data indicate that ancestral stress may in part limit behavioral and neuronal flexibility after a brain lesion (Metz et al., 2005b; Faraji et al., 2011). Such effects may be linked to miRNA regulation of neuroplasticity factors. Fortunately, the impact of adverse ancestral programming may be reduced by beneficial experiences, such as an enriched life style (McCreary et al., 2016), exercise (Duclos and Tabarin, 2016), massage therapy (Zucchi et al., 2014), mindfulness training (Carlson et al., 2004) and other therapies that mitigate elevated HPA axis activation. The present findings emphasize the impact of ancestral experiences in programming the capacity of neuroplasticity, risk and recovery from stroke.

## Author Contributions

JF, FCRZ and GASM designed the research; JF, MA and FCRZ performed the research; JF, NS and MA analyzed the data; JF, MA, PB, IK and GASM wrote the article.

## Conflict of Interest Statement

The authors declare that the research was conducted in the absence of any commercial or financial relationships that could be construed as a potential conflict of interest.

## Footnotes

^1^http://imagej.nih.gov/ij

^2^https://cutadapt.readthedocs.org/

^3^https://support.illumina.com/sequencing/sequencing_software/igenome.html
